# Correction: Humans perseverate on punishment avoidance goals in multigoal reinforcement learning

**DOI:** 10.7554/eLife.83998

**Published:** 2022-10-10

**Authors:** Paul B Sharp, Evan M Russek, Quentin JM Huys, Raymond J Dolan, Eran Eldar

**Keywords:** Human

Sharp PB, Russek EM, Huys QJM, Dolan RJ, Eldar E. 2022. Humans perseverate on punishment avoidance goals in multigoal reinforcement learning. *eLife* **11**:e74402. doi: 10.7554/eLife.74402.

Published 24 February 2022

Paul Sharp, the lead author, noticed two errors in the naming of variables in two the key equations in the article.

Equation 6 is incorrect. The equation in the published article read::

(6) $$\begin{array}{l} \left[\begin{array}{l} Q_{MB_{t+1}}(press=g)\\ Q_{MB_{t+1}}(press=j) \end{array}\right]\\ =\left[\begin{array}{ll} \beta_{MBreward}P(f=reward|press=g{)}_{t} & \beta_{MBpunish}P(f=punish|press=g{)}_{t}\\ \beta_{MBreward}P(f=reward|press=j{)}_{t} & \beta_{MBpunish}P(f=punish|press=j{)}_{t} \end{array}\right]\left[\begin{array}{c} 0\\ -1 \end{array}\right] \end{array}$$

The correction to equation (6) is as follows:

(6) $$\begin{array}{l} \left[\begin{array}{l} Q_{GP_{t+1}}(press=g)\\ Q_{GP_{t+1}}(press=j) \end{array}\right]\\ =\left[\begin{array}{ll} \beta_{GPreward}P(f=reward|a=press\;g{)}_{t} & \beta_{GPpunish}P(f=punish|a=press\;g{)}_{t}\\ \beta_{GPreward}P(f=reward|a=press\;j{)}_{t} & \beta_{GPpunish}P(f=punish|a=press\;j{)}_{t} \end{array}\right] \end{array}$$

Equation 8 also also had a slight error in how the action variable was named. It is now consistent with how all other equations reflect the action taken. The original equation in the article was:

(8) $$P\left(a=press\;j\right)=\frac{e^{Q_{Integrated}\left(press=j\right)}}{e^{Q_{Integrated}\left(press=j\right)}+e^{Q_{Integrated}\left(press=g\right)}}$$

The equation is now correction to read:

(8) $$P\left(press=\;j\right)=\frac{e^{Q_{Integrated}\left(press=j\right)}}{e^{Q_{Integrated}\left(press=j\right)}+e^{Q_{Integrated}\left(press=g\right)}}$$

We apologize for any confusion this might have caused.

