# Supplementary material for: Humans perseverate on punishment avoidance goals in multigoal reinforcement learning
Source: eLife. 2022 Feb 24;11:e74402. doi: 10.7554/eLife.74402 (PMC8912924; doi:10.7554/eLife.74402)
Supplement: Supplementary file 1. [file elife-74402-supp1.docx]

| **Task version 1** | mean | hdi_3% | hdi_97% | **Task version 2** | mean | hdi_3% | hdi_97% |
| --- | --- | --- | --- | --- | --- | --- | --- |
| MB Reward | -0.5 | -0.70 | -0.31 | MB Reward | -0.57 | -0.79 | -0.34 |
| MB Punishment | 0.13 | -0.07 | 0.32 | MB Punishment | 0.13 | -0.08 | 0.32 |
| GP Reward | -0.09 | -0.27 | 0.08 | GP Reward | -0.12 | -0.28 | 0.04 |
| GP Punishment | 0.42 | 0.26 | 0.59 | GP Punishment | 0.22 | 0.05 | 0.38 |
